# Vitamins and Microelement Bioavailability in Different Stages of Chronic Kidney Disease

**DOI:** 10.3390/nu9030282

**Published:** 2017-03-15

**Authors:** Magdalena Jankowska, Bolesław Rutkowski, Alicja Dębska-Ślizień

**Affiliations:** Department of Nephrology, Transplantology and Internal Medicine, Medical University of Gdańsk, Gdańsk 80-211, Poland; bolo@gumed.edu.pl (B.R.); adeb@gumed.edu.pl (A.D.-Ś.)

**Keywords:** vitamin, dialysis, malnutrition

## Abstract

Chronic kidney disease (CKD) predisposes one to either deficiency or toxic excess of different micronutrients. The knowledge on micronutrients—specifically water-soluble vitamins and trace elements—in CKD is very limited. Consequently, current guidelines and recommendations are mostly based on expert opinions or poor-quality evidence. Abnormalities of micronutrient resources in CKD develop for several reasons. Dietary restrictions and anorexia lead to an insufficient micronutrient intake, while diuretics use and renal replacement therapy lead to their excessive losses. Absorption is unpredictable, and metabolism impaired. Better understanding of the micronutrient needs of CKD patients could have an impact on many complications linked to vitamin and trace element disorders, including high mortality, increased risk of atherosclerosis, inflammation, oxidative stress, anemia, polyneuropathy, encephalopathy, weakness and fragility, muscle cramps, bone disease, depression, or insomnia. Here, we summarize the up-to-date knowledge on micronutrient resources in different stages of CKD, and share our experience with the assessment of micronutrient status.

## 1. Introduction

Overt deficiency of vitamins and essential minerals is extremely rare in developed societies. Nevertheless, it affects more than one-third of the world’s population, constituting an important healthcare burden, and has devastating consequences. Although malnutrition is an acknowledged problem in patients with chronic kidney disease (CKD), disordered micronutrient status is a much less recognized complication [1,2]. In most cases, micronutrient deficiency progresses slowly, and has clinical manifestations indistinguishable from that of CKD.

Recent evidence has shown that suboptimal levels of micronutrients—even well above those causing overt deficiency syndromes—may contribute to chronic complications such as cardiovascular disease, inflammatory status, or cancer. Thus, numerous complications of CKD may originate from imbalances in the bioavailability of micronutrients. Consequences of micronutrient malnutrition may include premature mortality, atherosclerosis, inflammation, oxidative stress, anemia, polyneuropathy, encephalopathy, weakness and fragility, muscle cramps, bone disease, depression, and insomnia.

CKD distinctly predisposes one to disorders of vitamins and trace elements. Many risk factors in CKD contribute mutually to malnutrition and inadequate micronutrient status. The main concern remains vitamin and trace element deficiency, although there is also a potential for the accumulation and unrecognized toxicity of micronutrients or their metabolites in individuals with compromised renal functions [3,4].

Micronutrient deficiency in CKD may develop due to specificity in dietary recommendations, comorbidities, concomitant medication, impaired intestinal absorption, changed metabolism, and excessive loss with urine or dialysate. The risk of micronutrient malnutrition also increases with age, and the age of the CKD population is rising [5,6,7,8].

## 2. Dietary Intake

Dietary intake of micronutrients is a potentially modifiable factor that may have a role in the prevention of nutritional deficiencies. Restrictions imposed on CKD patients aiming at reducing protein, phosphate, or potassium intake make it difficult to ensure adequate micronutrient content in the diet. There has been a considerable body of evidence gained in recent years showing that micronutrient deficiency in the diet is prevalent irrespectively of the modality of CKD treatment or customary food choices in different regions of the world. In the hemodialysis (HD) population of central Italy, even 90 percent of analyzed diets were outside the recommended intake limits for vitamins and minerals [9]. Even in well-nourished peritoneal dialysis (PD) patients from Guadalajara, Mexico, a high proportion of individuals had a vitamin intake not meeting the recommendations [10]. We recently performed a study comparing dietary vitamin intakes in patients with CKD not yet dialyzed (ND), undergoing PD or HD, and in successful kidney transplant recipients (KT) [11]. As could be anticipated, the KT group had the best dietary intake of nutrients overall. Nevertheless, the trend of insufficient vitamin intake in patients treated with dialysis was only partially reversed by successful KT. Dietary pattern was comparable between the ND and KT group, and between the HD and PD groups. Thus, the dialysis procedure seemed to be an important contributor influencing dietary intake. Aside from the modality of renal replacement therapy, other contributors may also influence micronutrient intake, including patients’ nutritional and inflammatory status, dialysis adequacy, and the preservation of residual renal function [10,12].

## 3. Absorption

Body homoeostasis of micronutrients also depends on their normal absorption in the gastro-intestinal tract. Most water-soluble vitamins are absorbed from the intestine in a specific carrier-mediated process. The expression of carriers for thiamine and folic acid has been shown to be significantly reduced in an animal model of CKD [13]. Accordingly, the intestinal absorption of riboflavin, pyridoxine, and biotin is also impaired in a CKD setting [14,15,16]. Thus, intestinal losses may constitute yet another mechanism of compromised micronutrient status in CKD.

## 4. Losses

Excessive loss of micronutrients may occur in all stages of CKD. In earlier stages of the disease, micronutrients are lost with urine due to the use of diuretics and/or insufficient reabsorption by specific transporters [17,18]. In end-stage-renal-disease (ESRD), vitamins or trace elements tend to be removed by dialysis, as those low-molecular-weight substances are not routinely present in HD and PD dialysis fluids. On the other hand, even their minute concentrations in dialysis fluid, sourced from water, could lead to increased blood levels, and to accumulation in a patient’s body.

There is limited data on the exact quantity of micronutrient dialysis-related losses. In addition, reported data may vary according to the usage of different techniques (high-flux or low-flux membrane, high volume convective therapy), different supplementation routine, or intra-individual differences [19,20]. We have performed a study assessing dialysis-related losses of thiamine diphosphate (TDP), an active form of vitamin B1. After the HD session, the concentration of TDP in whole blood decreased in all cases. The mean reduction of whole blood levels reached 40% after a single hemodialysis session (data unpublished). We also analyzed TDP peritoneal appearance rate, and peritoneal TDP losses. TDP losses through the peritoneal membrane were modest. However, we also found that in single cases, amounts lost via this route may exceed the daily recommended intake (DRI) for vitamin B1 (in press).

## 5. Medication

The potential interactions between concomitant medication and micronutrient metabolism or absorption should always be borne in mind. CKD patients may be exposed to drugs directly interfering with the micronutrient metabolism (e.g., methotrexate) or receive medications whose role in micronutrient homeostasis is less established (phosphate binders, ion exchange resins, or immunosuppressants). Recently, we have shown that prolonged steroid therapy as well as treatment with polyclonal anti-thymocyte globulin in patients after KT may have profound effects on their vitamin B6 status [21,22].

## 6. Measurements

Measurements of vitamin levels in blood, serum, or red blood cells are regarded as not thoroughly reliable for the assessment of suboptimal vitamin status, even in the general population. In the presence of CKD, interpretation of those measurements is further complicated for several reasons. Plasma levels of micronutrients poorly reflect their tissue stores. Vitamin concentrations in blood can vary significantly, and are influenced by hemodilution, inflammation, fasting, or RRT (renal replacement therapy). The most accurate measure of micronutrient status seems to be the determination of its functional deficiency, obtained by assessing the activities of enzymes that contain specific micronutrients in their prosthetic groups. However, even such an assessment is highly unreliable in CKD because the biological significance of abnormal levels is unclear. Plasma or serum levels of main micronutrients in relation to those found in healthy individuals are summarized in Table 1.

## 7. Micronutrients Most Affected in Different Stages of Chronic Kidney Disease

### 7.1. Ascorbic Acid

We observed ascorbic acid (AA) loss during a single dialysis session to be about 28% [27], which was consistent with previous estimations of 33% and 40%, respectively [28,29]. Recently, it has been shown that intradialytic losses may even reach 60% [19]. Given that, the losses of vitamin C during each dialysis session are huge. Moreover, the vitamin is also easily oxidized to dehydro-ascorbic acid during hemodialysis. Over the years, the indications for ascorbate supplementations have been formed very cautiously, and nowadays the recommended doses might not be optimal, and not meet the requirements. Perhaps the greatest concern is hyperoxalaemia, as oxalate is a major metabolite of AA [30]. The levels of oxalate in dialysis patients are twofold higher than normal, and after vitamin C supplementation might be even seven-fold higher. Plasma oxalate levels greater than 50 mcg/L might lead to its tissue accumulation (retina, joints, heart). However, recent advances in renal replacement therapy seem to be preventing such a complication.

### 7.2. Thiamine

Thiamine blood concentrations in CKD patients are either within reference range for healthy individuals or even increased. Nevertheless, the recent findings of high levels of thiamine antimetabolite (oxythiamine) in ESRD shed new light on understanding thiamine deficiency, which may not necessarily go in line with its low plasma values [31].

The classical symptoms of thiamine deficiency are cardiomyopathy or Wernicke’s encephalopathy. They are traditionally linked to chronic alcohol abuse. Nevertheless, this complication might occur—although rarely—as one of the causes of unexplained encephalopathy in dialysis patients. The literature has revealed many cases of Wernicke’s encephalopathy in this group [32,33,34]. In these cases, the clinical course of the disease was often different from the classical. Very often the main (or only) symptom was disturbed consciousness.

### 7.3. Pyridoxine

The vitamin B6 losses during dialysis are still controversial. There has been a reported 35% drop in pyridoxine concentration after a single dialysis session [20]. On the other hand, as the vitamin is tightly protein bound, its losses are regarded as rather moderate. The vitamin deficiency was not observed in patients receiving 50 mg pyridoxine after each dialysis session [35]. Conversely, in those CKD patients not receiving B6 supplementations, the B6 deficiency was found in 78%, 77%, 50%, and 34% of cases, respectively [36,37,38,39].

### 7.4. Folic Acid

Weak binding of folic acid (FA) to plasma proteins results in significant losses during each dialysis session. A 37% drop in plasma concentration has been described after a single procedure [20]. According to the available evidence, folate supplementation in a dose of 1 mg/day should prevent deficiencies in hemodialysis patients [35]. Interestingly, folate supplementation in a dose of 2 mg/day results in a five-fold increase in plasma concentration [40]. Overall, high plasma and erythrocyte levels following supplementation have been described—especially in PD patients [23]. High doses of folic acid were used to limit cardiovascular complications in ESRD patients, due to its effect on homocysteine methylation. However, the benefits of this practice have never been confirmed, and the risk for patients with vitamin B12 should be considered.

### 7.5. Zinc

Concentrations of zinc in the skin and hair of CKD patients have been low, while other tissues (including erythrocytes) may have concentrations comparable with healthy individuals, or even higher [41]. Plasma levels of HD patients are low, and supplements were given to this group in order to improve appetite, polyneuropathy, sexual functions, immunological response, or even lipid profile [42,43]. Unfortunately, results of a recent randomized study showed that low dose supplementation fails to correct low zinc status in the HD population [44]. The needs for zinc in CKD patients are not established. Interestingly, fractional urine losses are increased in this group, which may explain the low efficacy of the supplementation.

### 7.6. Selenium

In the general population, the prevalence of selenium deficiency is high due to its low dietary content. In CKD, the deficiency rises further, as selenium is malabsorbed and is lost with dialysis fluid and urine. Selenium deficiency leads to the development of Keshan disease, characterized by congestive cardiomyopathy. Despite the apparent risk for selenium deficiency in CKD [2,45], there is no recommendation regarding its routine supplementation.

## 8. Supplementation

The scarcity of evidence is reflected in the current guidelines and recommendations. CARI (Caring for Australasians with Renal Impairment) and the National Kidney Foundation (NKF/DOQI) released micronutrient recommendations only for children. Supplements of water-soluble vitamins are regarded as indicated in dialysis patients who are not receiving nutritional supplements. According to the recommendations, supplements of vitamins A, B12, and E are not indicated, since the dietary intake of these vitamins meets the recommended daily intakes. The European Best Practice Guidelines (EBPG) and the guidelines of the European Society for Clinical Nutrition and Metabolism (ESPEN) recommend exact doses of vitamin supplements, mostly corresponding with the recommended daily allowance for healthy adults. Nevertheless, a wide variation in supplement prescription has been observed across countries, and the practices regarding micronutrient supplementation are far from those recommended [1]. Health care providers may not regard water-soluble vitamins and trace elements as medically necessary, since limited data describes the impact of supplementation on clinical outcomes in this group of patients [24]. In 2004, a positive effect of micronutrient supplements on survival was reported in DOPPS (Dialysis Outcomes and Practice Pattern Studies) [1] and later confirmed in single-center prospective study [46]. However, it has never been confirmed in any prospective randomized study. The results of the up-to-date randomized trials performed in the CKD population have been invariably disappointing [44,47], and the necessity of supplementation has been challenged recently [48].

Presumably, a high proportion of CKD patients may be using supplements without medical consultation. Thus, nephrologists should make specific efforts to enquire about the use of dietary supplements in their patients. It is most important to ensure that they avoid dangerous practices, such as high doses of vitamin A, potassium, or copper.

## 9. Conclusions

The knowledge of micronutrient disorders in CKD is scarce, but has been receiving increased attention recently. Well-designed trials investigating micronutrient status in this population of patients are urgently needed. The ongoing DIET-HD study raises expectations for better understanding the micronutrient needs of CKD patients, and for establishing strategies to improve health outcomes with the use of dietary interventions in advanced kidney disease [49]. This is critical if micronutrient interventions are to be not only effective, but also targeted to those with the greatest need.

## Figures and Tables

**Table 1:** Summary of vitamin and microelement plasma or serum levels in chronic kidney disease (CKD) and different modalities of renal replacement therapy [2,23,24,25,26]. HD: hemodialysis; PD peritoneal dialysis.

	CKD 3–5	HD	PD
Zinc	↓	↓	↔
Selenium	↓	↓	↓
Manganese	↓	↓	↓/↔
Copper	↑	↑	↔
Thiamine	↓	↔/↑	↔
Riboflavin	?	↔/↓	↔/↓
Niacin	↔↔	↔	↑/↔
Pyridoxine	↑	↓	↓
Cobalamin	↔	↔	↔
Folic acid	↑	↔/↓	↔/↑
Ascorbic acid	↓	↓	↓

↓ decreased, ↑ increased, ↔ not changed (in comparison to healthy individuals).
